# Influence of the Coronavirus Disease 2019 Pandemic on Patients with ST-Segment Elevation Myocardial Infarction in Taiwan

**DOI:** 10.1155/2021/5576220

**Published:** 2021-04-14

**Authors:** Yuan-Heng Su, Kuan-Han Wu, Chih-Min Su, Chi-Yung Cheng, Cheng-I Cheng, Chia-Te Kung, Fu-Cheng Chen

**Affiliations:** ^1^Department of Emergency Medicine, Kaohsiung Chang Gung Memorial Hospital, Chang Gung University College of Medicine, Kaohsiung 83301, Taiwan; ^2^Division of Cardiology, Department of Internal Medicine, Kaohsiung Chang Gung Memorial Hospital, Chang Gung University College of Medicine, Kaohsiung 83301, Taiwan

## Abstract

The outbreak of the new coronavirus disease 2019 (COVID-19) has notably affected the medical system worldwide and influenced the health-seeking behavior of people while depleting medical resources, causing a delay in ST-elevation myocardial infarction (STEMI) management. In this single-center, retrospective cohort study, we compared the clinical pictures of nontransfer patients who presented to the emergency department directly and received primary percutaneous cardiovascular intervention (PPCI) from February 1 to April 30, 2020 (group 2, *N* = 28), with patients who received PPCI from February 1 to April 30, 2016–2019 (group 1, *N* = 130). A total of 158 patients with STEMI who received PPCI were included in the study. A decrease in the percentage of patients with door-to-balloon time <90 minutes was found in group 2 (64.3% vs. 81.5%, *p* = 0.044). The adjusted odds ratio was calculated using logistic regression, according to potential confounding factors such as age, sex, off-hours, and Killip class. An adjusted odds ratio of 2.45 (95% confidence interval, 1.1–6.0, *p* = 0.048) was reported for group 2. A decrease in the percentage of patients meeting the criteria of door-to-balloon time <90 minutes was demonstrated, and differences were revealed in the clinical pictures of patients with STEMI after the pandemic. While systemic factors contributed the most, improvements and adjustments in the protocols for managing patients with STEMI for better outcomes in the COVID-19 era have yet to be studied.

## 1. Introduction

Ever since the new coronavirus disease 2019 (COVID-19) emerged in Wuhan, China, it has spread rapidly across cities and countries, causing significant worldwide public health issues, and has been declared a pandemic [1]. Due to its highly contagious nature, which requires strict infection control measures to limit transmission, COVID-19 greatly impacts the global medical system by influencing the health-seeking behavior of people and depleting medical resources. Taiwan was expected to be a COVID-19 hotspot because of its proximity to China and the high number of to-and-fro flights to China. Nevertheless, only less than two thousand confirmed cases have been reported to date, owing to experienced public health professionals and well-trained personnel, who with early awareness and initiation of infection control strategies prevented major outbreaks.

However, strategies to control COVID-19 may lead to a delay in the treatment of patients with acute myocardial infarction. According to the American Heart Association guidelines for acute myocardial infarction, primary percutaneous cardiovascular intervention (PPCI) has been suggested for patients with ST-elevation myocardial infarction (STEMI). PPCI as the preferred method of reperfusion treatment for patients with STEMI is to be performed within 90 minutes upon arrival of a patient, not transferred from another hospital [2]. The shortening of door-to-balloon time is related to the survival rate and clinical outcome of patients [3]. Multiple strategies are implemented to reduce the door-to-balloon time; however, given the circumstances involving COVID-19 and the necessity of infection control strategies, patients admitted to hospitals via the emergency department (ED) or outpatient department require initial screening for COVID-19 prior to further examination and treatment to avoid in-hospital transmission of the disease. Thus, delay in diagnosis of patients with STEMI may occur owing to infection control strategies, including initial screening, isolation of patients presenting with fever and respiratory symptoms, and awaiting the COVID-19 laboratory test results.

Recently, many studies have revealed a decrease in the number of patients with STEMI during the COVID-19 pandemic, with an elevation in their mortality rate. For instance, a Spain-based study revealed a 40% decreased in PPCI performed [4], while studies in Italy revealed the reduction of admission rate of STEMI patients up to 48% [5, 6]. A similar scenario occurs in Austria and Hong Kong according to related studies [7, 8]. However, one study including nationwide German research revealed prolonged symptoms to medical service time and reported no significant difference in indicators for STEMI management during the current pandemic [9]. Hence, we aimed to investigate whether the clinical picture of patients with STEMI is different due to the prevailing COVID-19 pandemic and the corresponding precautionary measures.

## 2. Materials and Methods

### 2.1. Study Design

This retrospective cohort study was based on the database review of patients who presented to our ED with STEMI excluding transferred patients. Patients were analyzed before and during the pandemic. We attempted to correlate patient outcomes with certain parameters statistically. All patient and physician data were deidentified, and the hospital's Institutional Review Committee on Human Research (202000911B0) approved the study.

### 2.2. Study Setting and Population

This is a single-center study, based in the Kaohsiung Chang Gung Memorial Hospital, which is a medical center in southern Taiwan with over 2,500 inpatient beds and 5,500 employees. The institution provides 24/7 PPCI services for patients with STEMI. Over 150 patients with STEMI have been treated annually since 2001. It is one of the largest medical centers in southern Taiwan, and approximately one-third of its patients are transfers from nearby regional hospitals and clinics. Prehospital ECG transmission and prehospital PPCI lab are activated on patient transferring between hospitals (cooperation/network), while emergency physician PPCI lab activation was also performed.

Protocols to reduce the risk of transmission of COVID-19 were implemented [10]. We evaluated and categorized the patients with respect to their risk of being infected, based on the history of fever, respiratory symptoms, travel, occupation, cluster, and contact information provided. The patients were then separated into different zones according to their risk level in order to limit the contaminated areas. Further examinations were performed after a primary survey for risk evaluation of COVID-19. Based on clinical symptoms and electrocardiogram (ECG) findings, cardiologists were consulted for patients with STEMI to initiate the protocol in order to perform PPCI.

In our study, we included patients who were diagnosed with acute STEMI according to ECG findings and have received PPCI from February to April 2020, while those who had received PPCI during the same months in the past 4 years comprised the control group. Exclusion criteria included patients referred from other medical providers, patients with out-of-hospital cardiac arrests, patients not receiving PPCI due to refusal of the procedure, or deaths before the procedure.

### 2.3. Data Acquisition

Baseline characteristics; vital signs upon arrival; laboratory data; angiographic findings; time intervals including door-to-ECG time, door-to-balloon time, and door-to-balloon time <90 minutes; time of patient arrival in regular hours or off-hours (regular hours were Monday to Friday, 08 : 00 to 17 : 00, while off-hours were Monday to Friday, 17 : 01 to 07 : 59, weekends, and public holidays); Killip class (evaluation of patient severity according to physical examination and degree of heart failure for prediction of mortality risks [11]); and outcome parameters such as postprocedural thrombolysis in myocardial infarction (TIMI)-3 flow (grade 3 represents normal flow after thrombolysis [12, 13]) and in-hospital mortality were acquired. Anonymization and deidentification of data were performed prior to statistical analysis.

### 2.4. Statistics

Regarding the continuous variables, mean ± standard deviation and Student's *t*-test were used for presentation and analysis, respectively. For categorical variables, numbers and percentages were presented using the chi-square test for analysis. Evaluation and analysis of each time interval between the two groups were performed using the Mann–Whitney *U* test as the time variables were not normally distributed and nonparametric tests were preferred. For evaluation of the association between the two groups, logistic regression was used to calculate the adjusted odds ratio, and potential confounding factors including age, sex, off-hours, and Killip class were taken into account. Hosmer–Lemeshow goodness-of-fit model and stepwise regression analysis were used. *p* values ≤ 0.05 (two-tailed Student's *t*-test) were considered to be statistically significant. All analyses were conducted using SPSS for Windows (version 25.0; SPSS, Chicago, IL, USA).

## 3. Results

### 3.1. Patient Inclusion

The study included 158 patients with STEMI who had visited our ED. The two groups included patients with STEMI who received PPCI from February 1 to April 30, 2020 (group 2, *N* = 28) and patients who received PPCI from February 1 to April 30 in 2016–2019 (group 1, *N* = 130).

### 3.2. Baseline Information

The baseline characteristics, vital signs upon arrival, and laboratory data are shown in Table 1. Between the two groups of patients with STEMI, there were no significant differences regarding age, sex, body mass index, diabetes mellitus, hypertension, dyslipidemia, current smoker or previous myocardial infarction status, vital signs, laboratory data, off-hours, and Killip class.

### 3.3. Angiographic Features and Clinical Outcome

Table 2 presents the angiographic features of the patients, including symptom-to-door time, door-to-ECG time, door-to-balloon time, the number of coronary arteries narrowed, and stenting. In order to evaluate patient outcomes, the postprocedural TIMI-3 flow, length of hospital stay, and in-hospital mortality were analyzed. For most time series parameters including symptom-to-door time, symptom-to-ECG time, door-to-balloon time, and reperfusion time, there were no significant differences between the two groups. Nevertheless, a comparison of the percentage of patients who met the criteria of the door-to-balloon time <90 minutes revealed a notable decrease during the COVID-19 pandemic (64.3% in group 2 and 81.5% in group 1, *p* = 0.044). We also compared the clinical outcomes of patients between the two groups; however, there were no significant differences in postprocedural TIMI-3 flow, length of hospital stay, and in-hospital mortality. In order to analyze the association of door-to-balloon time <90 minutes between groups 1 and 2, logistic regression analysis was performed and the odds were estimated. We used group 1 as the reference and calculated the adjusted odds ratio based on potential confounding factors including age, sex, off-hours, and Killip class; the results are shown in Table 3. The adjusted odds ratio of group 2 was 2.45 (95% confidence interval, 1.1–6.0, *p* = 0.048), which indicated that the percentage of door-to-balloon time <90 minutes in group 2 was significantly reduced.

**Figure 1:**
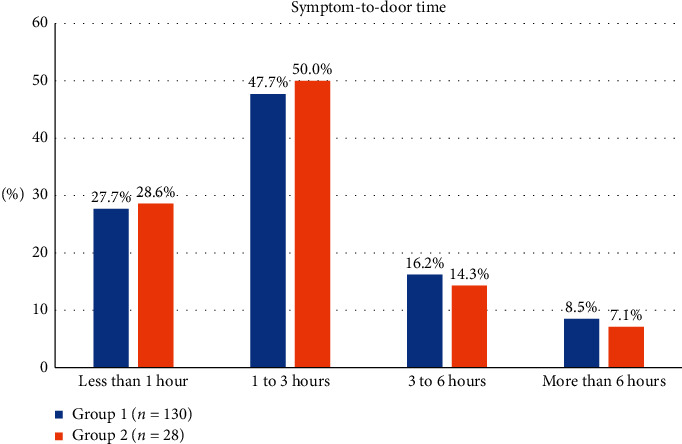
Distribution of symptom-to-door time between the two groups.

**Figure 2:**
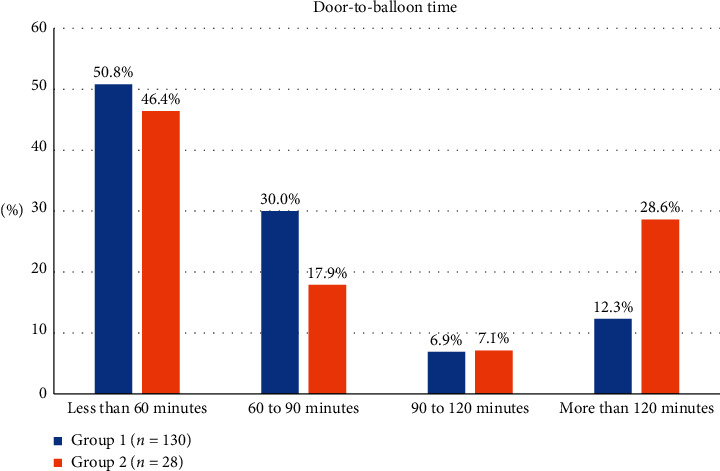
Distribution of time sequence between the two groups. The percentage of door-to-balloon time more than 120 minutes in group 2 was greater than that in group 1.

The distribution of symptom-to-door time between the two groups was generally equal, as shown in Figure 1. For door-to-balloon time, the distribution of time sequence is presented in Figure 2, revealing that the percentage of door-to-balloon time >120 minutes in group 2 was greater than that in group 1.

## 4. Discussion

The outbreak of COVID-19 has had a significant impact on the medical system and is still expanding. Our research found that COVID-19 impacts patients with STEMI by reducing the number of patients with door-to-balloon time <90 minutes. This may be due to the COVID-19 infection control strategies.

These strategies included pretriage for measuring temperature; taking travel, occupation, and contact history; checking immigration records [14]; and separating the patients who are likely to have COVID-19 but require urgent treatment into zones with fully equipped staff. For patients with STEMI with the risk of COVID-19, a swab screening was performed, and the results were acquired before the patients were sent to the catheterization lab. The above protocols are likely to increase the time patients spend in the ED before receiving PPCI, thus decreasing the percentage of patients with door-to-balloon time <90 minutes.

The protocols followed for patients with STEMI in other countries impacted by COVID-19 vary. In the United States, PPCI remains the treatment of choice for patients with STEMI in PCI-capable hospitals; however, fibrinolysis management may be conducted in non-PCI-capable hospitals or under certain circumstances [15]. Nevertheless, there have been studies that have reported an estimated decrease of 38% in PCI activations in patients with STEMI in the United States owing to patients avoiding medical care due to the concern of being infected with COVID-19 in hospital and an increase in the pharmacological management of STEMI [16]. On the other hand, the Canadian Association of Interventional Cardiology has issued guidance regarding cardiovascular diseases and their management according to the response level against COVID-19 as it escalates. As the response levels escalate from low to high, management of STEMI varies from planned PPCI with personal protective equipment (PPE) and N95 masks following a COVID-19 rapid test to fibrinolysis therapy only [17].

For countries severely affected by COVID-19, the impact on STEMI care has been elaborated in several studies. A study based in Spain revealed a 40% decrease in PPCI performed on patients with STEMI and additionally a delay in door-to-balloon time, attributed to fewer health-seeking behaviors, and a significant reduction in healthcare services provided [4]. While Italy has been one of the most affected countries in the world, research has shown that the admission rate of patients with STEMI significantly reduced to 48% during the pandemic and national lockdown [5, 6]. A nationwide retrospective study in Austria also reported a significant decrease in patients admitted with acute coronary syndromes, which is possibly due to patient- and system-related reasons [7]. In Hong Kong, delays in treatment and potential undesirable outcomes were reported in a single-center cohort study, which was probably due to behavioral changes among patients and staff due to the pandemic and strict infection control measures [8]. However, a national cohort study including 41 hospitals providing PPCI services in Germany reported a different clinical picture of STEMI care during the COVID-19 pandemic. A mild decrease of 12.6% was observed in the number of patients with STEMI treated during the pandemic [9]. A significant increase in cath-to-puncture time was stated in the study, indicating that infection control precautions were being taken. Other parameters including symptom-to-contact time, contact-to-door time, door-to-balloon time, and hospital mortality during the pandemic showed no significant differences compared to the years preceding the COVID-19 outbreak, indicating no quality impairments in STEMI care during the pandemic. Given the clinical picture of different countries, we found that the number of patients with STEMI who visited hospitals for medical care decreased and door-to-balloon time increased in countries that were severely affected by COVID-19, while these changes were less extensive in countries that were relatively stable.

Owing to the COVID-19 pandemic, there have been changes in the health-seeking behavior of people; moreover, the sustainability of healthcare systems is being challenged. Our study findings elaborated the impact of COVID-19 on the protocols and management of patients with STEMI. With the exception of some countries, treatment delays have been reported globally. However, there are some improvements that could be implemented for potentially better prognosis of patients with STEMI during the pandemic. In our study, patients with prolonged door-to-balloon time were mostly suspected to have COVID-19 and their screening results were awaited. Nevertheless, COVID-19 could not be ruled out even if the first test result was negative; thus, adequate personal protection equipment (PPE) is still mandatory for all cardiovascular catheterization laboratory staff. For patients with STEMI who were suspected to have COVID-19 infection, PPCI could be performed under strict infection control protocols using protective equipment before the results of the COVID-19 test were received if healthcare resources are sufficient. Considerations for infection control have been reported in several studies, including the rescheduling of nonurgent PPCIs, minimizing the staff during an urgent PPCI, using a negative pressure room for the procedure, properly using PPE, and using noninvasive fibrinolytic therapy if PPCI is not available [18–20]. Treatment of STEMI patients in the era of COVID-19 has been significantly altered; hence, more research on different scenarios and factors must be conducted for the elaboration of ideal management with respect to the diversity in circumstances [21].

## 5. Limitations

Several limitations were noted in our study. First, given that our study was retrospective and the statistics showed that there were no significant differences between the baseline conditions of the two groups of patients, there were still some confounding factors that were beyond control and may have been ignored. Second, the sample size we obtained was relatively small, which may cause some statistical data and power problems. However, Taiwan was affected by the pandemic for a short period of time and most medical services have almost returned to normal. Therefore, it is difficult to include more patients affected by COVID-19. Third, this research was conducted at a single center. The strategies employed at other medical centers for dealing with COVID-19 may not be exactly the same; moreover, the protocols for patients with acute myocardial infarction may also be different. Therefore, the findings of our study cannot be generalized.

## 6. Conclusions

A decrease in the percentage of patients with door-to-balloon time <90 minutes, mostly attributed to systemic factors, was found in our study. The implementation of COVID-19 screening and infection control approaches for new patients presenting to the ED and outpatient department may prolong the time before patients with STEMI get treated. However, the necessity of strict infection control measures and outcomes of the modes of reperfusion by PPCI or fibrinolytic therapy require further investigation.

## Figures and Tables

**Table 1:** Baseline characteristics, vital signs, and laboratory data.

Variables	Group 1	Group 2	*p* value
	February to April 2016–2019 (*n* = 130)	February to April 2020 (*n* = 28)	
Age (years)	60.9 ± 12.8	59.3 ± 11.9	0.541
Male gender	102 (78.5)	24 (85.7)	0.450
Body mass index (kg/m^2^)	25.5 ± 4.3	26.0 ± 3.6	0.472
Diabetes mellitus	45 (34.6)	10 (35.7)	1.000
Hypertension	93 (71.5)	18 (64.3)	0.496
Dyslipidemia	110 (84.6)	24 (85.7)	1.000
Current smoking	68 (52.3)	15 (53.6)	1.000
Previous myocardial infarction	21 (16.2)	2 (7.1)	0.374
Systolic blood pressure^a^	138 ± 35	129 ± 34	0.272
Heart rate^a^	79 ± 20	78 ± 20	0.825
White blood cell count (k/mm^3^)^a^	10.2 (8.5–12.4)	11.0 (8.6–12.8)	0.722
Hemoglobin (g/dL)^a^	14.6 (13.2–15.5)	14.3 (12.3–15.6)	0.835
Platelet count (k/mm^3^)^a^	225 (186–271)	243 (193–294)	0.485
Sugar (mg/dL)^a^	162 (131–209)	152 (135–227)	0.531
Creatinine (mg/dL)^a^	1.1 (0.9–1.3)	1.1 (1.0–1.4)	0.298
AST (U/L)^a^	33 (26–45)	25 (20–47)	0.164
Troponin-I (ng/mL)^a^	0.06 (0.01–0.53)	0.03 (0.01–0.14)	0.532
Off-hour	96 (73.8)	22 (78.6)	0.848
Killip class II–IV^a^	45 (34.6)	10 (35.7)	1.000

Data are expressed as mean ± SD or *n*(%) or median (25^th^-75^th^ percentile). ^a^The data were measured upon presentation. ^*∗*^*p* < 0.05. AST, aspartate aminotransferase.

**Table 2:** Angiographic feature, results, and clinical outcomes.

Variables	Group 1	Group 2	*p* value
	February to April 2016–2019 (*n* = 130)	February to April 2020 (*n* = 28)	
Symptom-to-door time (hours)	1.8 (0.9–3.0)	1.3 (0.9–2.7)	0.532
Door-to-ECG time (minutes)	6 (4–8)	5 (4–9)	0.741
Door-to-cath room time (minutes)	38 (32–56)	43 (33–124)	0.532
Reperfusion time (minutes)	18 (14–23)	19 (15–27)	0.514
Door-to-balloon time (minutes)	59 (48–78)	62 (52–150)	0.243
Door-to-balloon time < 90 minutes	106 (81.5)	18 (64.3)	0.044^*∗*^
Postprocedural TIMI-3 flow	126 (96.9)	27 (96.4)	0.892
Stenting	124 (95.4)	28 (100)	0.246
Multivessel disease (≥2 vessels)	87 (66.9)	19 (67.9)	0.924
Length of hospital stay (days)	4.6 (3.5–6.8)	3.7 (3.0–5.5)	0.699
LVEF	55 (46–55)	59 (46–69)	0.858
In-hospital mortality	11 (8.5)	1 (3.6)	0.376

Data are expressed as mean ± SD or *n* (%) or median (25th–75th percentile). ECG: electrocardiography; LVEF: left ventricular ejection fraction; TIMI: thrombolysis in myocardial infarction. ^*∗*^*p* < 0.05.

**Table 3:** The association between groups 1 and 2 and door-to-balloon time less than 90 minutes by logistic regression analysis.

Outcome	Group 1	Group 2	95% CI	*p* value
	Reference	aOR		
Door-to-balloon time <90 minutes	1	2.45	1.1–6.0	0.048

aOR, adjusted odds ratio; 95% CI, 95% confidence interval. The adjusted odds ratio was calculated based on potential confounding factors including age, sex, off-hours, and Killip class.

## Data Availability

The data used to support the findings of this study are available from the corresponding author upon request.
